# Impact of numerical variation, allele burden, mutation length and co-occurring mutations on the efficacy of tyrosine kinase inhibitors in newly diagnosed FLT3- mutant acute myeloid leukemia

**DOI:** 10.1038/s41408-020-0318-1

**Published:** 2020-05-04

**Authors:** Iman Abou Dalle, Ahmad Ghorab, Keyur Patel, Xuemei Wang, Hyunsoo Hwang, Jorge Cortes, Ghayas C. Issa, Fevzi Yalniz, Koji Sasaki, Dai Chihara, Allyson Price, Tapan Kadia, Naveen Pemmaraju, Naval Daver, Courtney DiNardo, Farhad Ravandi, Hagop M. Kantarjian, Gautam Borthakur

**Affiliations:** 1 Department of leukemia, The University of Texas MD Anderson Cancer Center, Houston, TX USA; 2 Department of hemopathology, The University of Texas MD Anderson Cancer Center, Houston, TX USA; 3 Department of biostatistics, The University of Texas MD Anderson Cancer Center, Houston, TX USA

**Keywords:** Medical research, Acute myeloid leukaemia

## Abstract

*FLT3*-ITD mutations in newly diagnosed acute myeloid leukemia (AML) are associated with worse overall survival (OS). *FLT3*-ITD diversity can further influence clinical outcomes. Addition of FLT3 inhibitors to standard chemotherapy has improved OS. The aim of this study is to evaluate the prognostic impact of FLT3 diversity and identify predictors of efficacy of FLT3 inhibitors. We reviewed prospectively collected data from 395 patients with newly diagnosed *FLT3*-ITD mutant AML. 156 (39%) patients received FLT3 inhibitors combined with either high or low intensity chemotherapy. There was no statistically significant difference in clinical outcomes among patients treated with FLT3 inhibitors based on FLT3 numerical variation (*p* = 0.85), mutation length (*p* = 0.67). Overall, the addition of FLT3 inhibitor to intensive chemotherapy was associated with an improved OS (HR = 0.35, 95% CI: 0.24–0.5, *p* = 0.0005), but not in combination with lower intensity chemotherapy (HR = 0.98, 95%CI: 0.7–1.36, *p* = 0.85). A differential effect of FLT3 inhibitor on OS was more pronounced in younger patients with FLT3 allelic ratio ≥0.5 (HR = 0.41, 95% CI: 0.25–0.66, *p* < 0.001), single ITD mutation (HR = 0.55, 95% CI: 0.34–0.88, *p* = 0.01), diploid cytogenetics (HR = 0.52, 95% CI: 0.35–0.76, *p* = 0.001), NPM1 co-mutation (HR = 0.35, 95% CI: 0.19–0.67, *p* = 0.001). Our analysis identifies predictors of survival among diverse FLT3 related variables in patients treated with FLT3 inhibitor.

## Introduction

Fms-like tyrosine kinase (*FLT3*) mutations including internal tandem duplications (ITDs) of the juxtamembrane domain (JMD), first described in 1996, constitute 25% of all newly diagnosed AML patients^1–4^. *FLT3*-ITD mutations lead to constitutive auto-phosphorylation of the receptor tyrosine kinase, resulting in an increased proliferation and survival of leukemic cells^5^. Insertions of as few as 15 base pairs (bp) can disrupt the auto-inhibitory receptor function^6^. *FLT3*-ITD mutations in newly diagnosed AML are associated with higher white cell count, increased relapses, and worse overall survival (OS)^2,7^. Both NCCN and ELN guidelines incorporate *FLT3*-ITD mutation in risk stratifying patients based on the allelic burden, as well as *NPM1* co-mutation^8,9^. The mutational diversity of *FLT3*-ITD such as mutant-to-wild type allelic ratio, insertion site, mutation length, and the co-occurring mutations seem to determine the prognostic value of *FLT3*-ITD in patients with newly diagnosed *FLT3*-mutant AML^10–15^. A number of tyrosine kinase inhibitors (TKI) were developed in order to disrupt the oncogenic signaling initiated by FLT3^16^. The addition of FLT3 inhibitors to chemotherapy in the frontline setting has improved OS^17^.

The information regarding the prognostic impact of *FLT3-*ITD mutation diversity in the context of FLT3 inhibitor therapy is of critical importance, however it has not been fully elucidated. The aim of this study is to evaluate the impact of numerical variation, mutation length, allelic burden, and co-occurring mutations on clinical outcomes and to identify predictors of efficacy of TKI in combination with chemotherapy, in patients with newly diagnosed *FLT3-*ITD mutant AML.

## Methods

### Patients

We searched our prospectively collected database of all patients with newly diagnosed AML, treated at MD Anderson cancer center from January 2000 until December 2017, for *FLT3*-ITD mutant AML (excluding acute promyelocytic leukemia and core binding factor AML). Patients were categorized into four groups based on the type of treatment received, as follows: high intensity chemotherapy with or without TKI, low intensity chemotherapy with or without TKI. High intensity chemotherapy included high dose cytarabine, idarubicin with or without a second nucleoside analog (i.e., cladribine, or fludarabine, or clofarabine), whereas low intensity chemotherapy included either hypomethylating agents (HMA) (i.e., 5-azacitidine or decitabine) or low dose cytarabine. Baseline variables including *FLT3*-ITD numerical variation, allelic burden, mutation length, and co-occurring mutations as well as clinical outcomes were collected and analyzed.

This study was performed in accordance with the Declaration of Helsinki. The Institutional Review Board approved the collection of data and a waiver of informed consent was granted for this chart review study.

### Clinical endpoints

The primary endpoint of this study is to evaluate the impact of *FLT3*-ITD mutational diversity including numerical variation, mutation insertion length, allelic burden, and co-occurring mutations on OS and relapse free survival (RFS) in newly diagnosed patients with *FLT3*-ITD AML particularly in the context of high or low intensity therapies and addition of a FLT3 inhibitor. The secondary endpoints are to assess the important predictors of FLT3 inhibitor efficacy in each of *FLT3*-ITD diverse variables.

### FLT3-ITD mutation analysis

PCR-based DNA analysis was performed to detect *FLT3*-ITD and codon 835/836 point mutation in the *FLT3* gene as per institutional protocol. A multiplex PCR using fluorescently labeled primers was performed, followed by detection and sizing of PCR products using capillary electrophoresis. For detecting point mutations in codons 835/836, a restriction enzyme digestion of the PCR products was performed prior to capillary electrophoresis. The lower limit of detection (analytical sensitivity) of the assay is ~1% of mutant DNA in a background of wild type DNA. The mutant allelic burden was calculated as the ratio of area under the peak of mutant over wild-type *FLT3*.

### Response and outcome definitions

Complete remission was defined as the presence of <5% blasts in the bone marrow, with ≥1 × 10^9^/L neutrophils, and ≥100 × 10^9^/L platelets in the peripheral blood. RFS was defined as the time from the date of response to the date of relapse or death due to any cause (censored at last follow-up date). OS was defined as the time from the date of diagnosis until death due to any cause (censored at last follow-up date).

### Statistical methods

Patients’ characteristics were summarized using descriptive statistics including median (range) for continuous variables and frequency (%) for categorical variables. To compare groups, the Fisher’s exact test and Wilcoxon rank-sum test were conducted for categorical and continuous variables, respectively. The Kaplan Meier method was used to estimate RFS and OS, and the log-rank test was performed to compare the time to events. The Cox proportional hazards regression model was fit to evaluate the association between clinical factors and survival outcomes. For the multivariate Cox model, all the variables significant at 0.05 in the univariate analysis were included, and backward stepwise elimination was applied to select variables for the final model. To identify a differential effect of TKI on outcomes per subgroups, interaction analysis using the Cox model was conducted, where an interaction term was composed of TKI and a subgroup variable. All computations were carried out in R version 3.5.1 and GraphPad prism version 7.

## Results

### Baseline characteristics

We identified 395 adult patients with FLT3-ITD mutant AML among the consecutive cohort of newly diagnosed AML. The median age for all patients is 61 years (range, 17–89 years). Of them, 223 (56%) patients were treated with high intensity chemotherapy, including 94 patients who received TKI (24%) and 129 (32%) patients who did not receive TKI. The other 172 (44%) patients were treated with low intensity chemotherapy, including 62 (16%) patients who received TKI and 110 (28%) patients who did not receive TKI. Baseline characteristics of patients in each group are summarized in Table 1 and S1. In the high intensity chemotherapy group, patients who received TKI were younger with a median age of 52 years (range, 20–78 years) compared with those who did not receive TKI, median 55 years (range, 17–82 years) (*p* = 0.02). The median WBC count was also significantly higher in patients who did not receive TKI (median 14.2 × 10^9^/L, range, 0.8–191 × 10^9^/L versus 8.5 × 10^9^/L, range, 0.5–378 × 10^9^/L) (*p* = 0.03). Patients who were treated with TKI were more likely to receive a second nucleoside analog (i.e., cladribine, or fludarabine, or clofarabine) in addition to idarubicin, cytarabine compared with patients who did not receive TKI (66% versus 19%, *p* < 0.001 respectively). The rate of subsequent allogeneic hematopoietic stem cell transplantation (allo-HSCT) in first CR was 57% in patients who received TKI versus 33% in patients who did not receive TKI (*p* < 0.001).

**Table 1:** Baseline characteristics.

Variables	No. (%), median [range] *n* = 223		*p* value	No. (%), median [range] *n* = 172		*p* value
	High intensity chemotherapy			Low intensity chemotherapy		
	With TKI (*n* = 94)	Without TKI (*n* = 129)		With TKI (*n* = 62)	Without TKI (*n* = 110)	
Age, years	52 [20–78]	55 [17–82]	0.02	71.5 [52–86]	71 [21–89]	0.69
Male	36 (38.3)	60 (46.5)	0.27	31 (50)	65 (59)	0.27
WBC, ×10^9^/L	8.45 [0.5–378]	14.2 [0.8–191]	0.03	8 [0.2–164.5]	11.7 [0.4–186.5]	0.14
LDH, IU × 10^−3^	1 [0.37–8.77]	0.99 [0.37–11.15]	0.46	0.98 [0.28–10.3]	1.08 [0.23–42]	0.41
BM blasts, %	54 [0–98]	43 [0–99]	0.50	26.5 [0–98]	46 [0–98]	0.38
Diploid CG	70 (74.5)	87 (69)	0.45	42 (75)	75 (72.8)	0.85
Complex CG	5 (5.3)	5 (3.9)	0.75	3 (5.1)	7 (6.4)	>0.99
ELN 2017 subgroup						
1	18 (19.2)	14 (11)	0.05	9 (14.5)	17 (15.4)	0.69
2	46 (48.9)	82 (63.5)		35 (56.5)	66 (60)	
3	30 (31.9)	30 (23.2)		18 (29)	25 (22.7)	
*FLT3*-ITD size	53 [8–195]	53 [17–227]	0.86	54 [7–207]	45 [2–195]	0.38
*FLT3*-ITD ratio	0.56 [0.01–2.46]	0.35 [0.01–7.7]	0.21	0.64 [0.01–2.56]	0.32 [0.01–6.11]	0.002
*FLT3*-ITD number						
1	45 (47.8)	104 (81.9)	<0.001	28 (45.2)	77 (70)	<0.001
2	15 (15.9)	15 (11.8)		14 (22.6)	22 (20)	
3	34 (36.2)	8 (6.3)		20 (32.3)	11 (10)	
*FLT3* D835	6 (6.4)	10 (7.7)	0.80	9 (14.5)	7 (6.4)	0.10
*NPM1*	45 (51.1)	34 (45.3)	0.53	32 (54.2)	31 (39.3)	0.09
*IDH1/2*	16 (22.9)	13 (26)	0.83	13 (22.8)	13 (22.8)	0.40
*DNMT3A*	18 (28.1)	9 (27.3)	>0.99	22 (44)	3 (6.4)	<0.001
No. co-mutations						
0	22 (23.7)	66 (58.4)	<0.001	11 (18)	53 (52)	<0.001
1	33 (35.5)	29 (25.7)		17 (27.9)	25 (24.5)	
2	20 (21.5)	15 (13.3)		19 (31.2)	19 (18.6)	
≥3	18 (19.4)	3 (2.6)		14 (22.9)	5 (4.9)	
Treatment regimen						
Doublet	32 (34)	105 (81.4)	<0.001			
Triplet	62 (66)	24 (18.6)				
HMA-based				51 (82.3)	44 (40)	<0.001
LDAC-based				9 (14.5)	49 (44.5)	
TKI						
Quizartinib	3 (3.2)	–		13 (21)	–	
Sorafenib	91 (96.8)	–		40 (64.5)	–	
Midostaurin	**–**	–		8 (12.9)	–	
Axitinib	–	–		1 (1.6)	–	
AlloHSCT	54 (57.5)	43 (33.3)	<0.001	8 (13)	9 (8.2)	0.43

In patients who were treated with low intensity chemotherapy, *FLT3*-ITD ratio was significantly higher in patients receiving TKI with a median of 0.64 (range, 0.01–2.56) versus 0.32 (range, 0.01–6.11) in patients who did not receive TKI (*p* = 0.002). Patients who received TKI were more likely to receive HMA (i.e., 5-azacitidine, or decitabine as opposed to LDAC) compared with patients who did not receive TKI (82% versus 40%, *p* < 0.001 respectively).

### Survival outcomes

#### High intensity chemotherapy

**Fig. 1:**
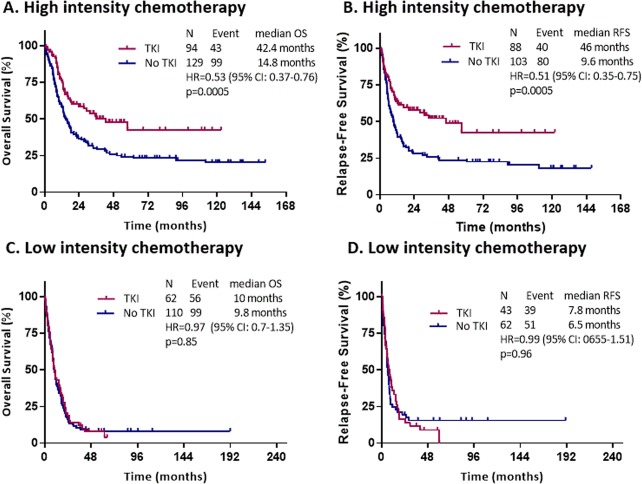
Survival outcomes by TKI use. **a**, **b** Overall survival and relapse free survival for all patients treated with high intensity chemotherapy by TKI use, without censoring to allogeneic hematopoietic stem cell transplantation. **c**, **d** Overall survival and relapse free survival for all patients treated with low intensity chemotherapy by TKI usage, without censoring to allogeneic hematopoietic stem cell transplantation.

With a median follow-up of 66 months, patients who were treated with high intensity chemotherapy with TKI had a median OS of 42.4 months (95% CI: 23.7 months -not reached) compared with 14.8 months (95% CI: 12.1–20.4 months) for patients treated without TKI (HR = 0.53, 95% CI: 0.37–0.76, *p* = 0.0005) (Fig. 1a). The median RFS was 46 months (95% CI: 16.7 months -not reached) versus 9.6 months (95% CI: 7.1–14.5 months) respectively (HR = 0.51, 95% CI: 0.35–0.75, *p* = 0.0005) (Fig. 1b). After censoring for allo-HSCT, the median OS was not statistically different between the TKI and no TKI groups (median OS, 15.9 versus 12.5 months, *p* = 0.07, respectively). Although statistically not significant, the 3-year RFS was 41.8% versus 21.9% respectively for patients who received TKI compared with those who did not receive TKI (Fig. S1, A-B).

#### Low intensity chemotherapy

With a median follow-up of 61.4 months, patients who were treated with low intensity chemotherapy with TKI had a median OS of 10 months (95% CI: 8.1–16.7 months) compared with 9.8 months (95% CI: 8.4–12.4 months) for patients who did not receive TKI (HR = 0.97, 95% CI: 0.7–1.35, *p* = 0.85) (Fig. 1c). The median RFS was 7.8 months (95% CI: 5.5–14.7 months) versus 6.5 months (95% CI: 5.7–8.2 months) respectively (HR = 0.99, 95% CI: 0.65–1.51, *p* = 0.96) (Fig. 1d). Due to the imbalance in the lower intensity chemotherapy regimen (HMA versus LDAC), we analyzed OS only for patients who were treated with HMA with or without TKI. There was also no statistical difference in OS between the two groups (HR = 0.89, 95% CI: 0.58–1.37, *p* = 0.62) (Fig. S2). Interestingly, patients who were treated with HMA and a potent FLT3 inhibitor, quizartinib (*n* = 10), had better OS with a median of 20.4 months compared with 11.4 months for those who were treated with HMA alone (HR = 0.44, 95% CI: 0.20–0.96, *p* = 0.035). This benefit was not shown with the addition of sorafenib to HMA (HR = 1.18, 95% CI: 0.75–1.88, *p* = 0.47) (Fig. S3, A-B).

### Prognostic significance of *FLT3*-ITD numerical variation

**Fig. 2:**
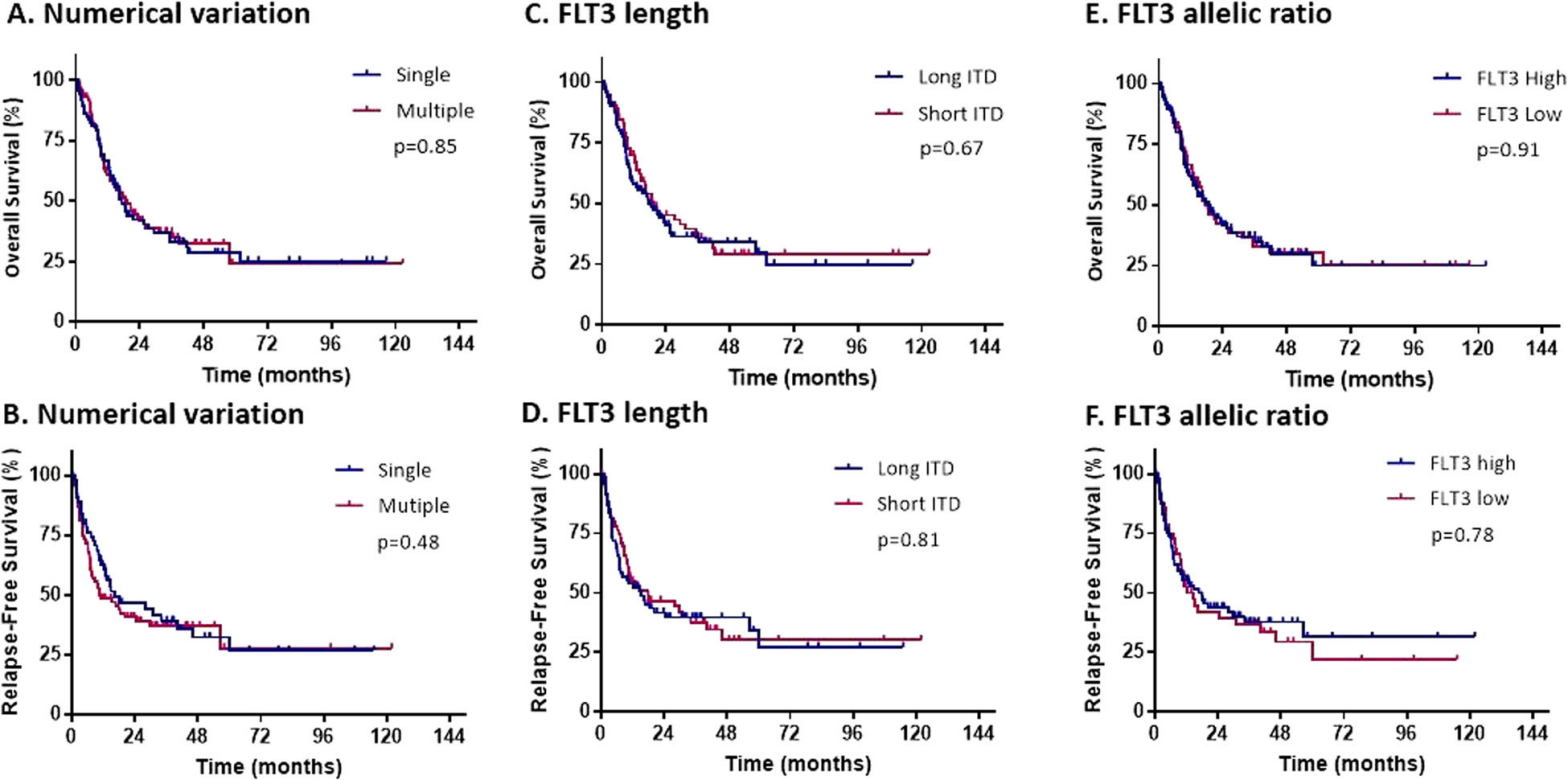
Survival outcomes by *FLT3* numerical variation, length and allelic ratio. Overall survival and relapse free survival for all patients receiving FLT3 inhibitors **(a**, **b)** based on FLT3-ITD numerical variation (single versus multiple) **(c**, **d)** *FLT3-ITD* mutation length (Long versus Short) **(e**, **f)** FLT3-ITD allelic ratio (high ≥0.5, low <0.5).

Among all patients included in this analysis, 256 (65%) had a single *FLT3* mutation, 66 (17%) had two mutants, and 73 (18%) had three mutants. Among patients who were treated with TKI, a single *FLT3* mutation, two mutants, and three mutants were present in 73 (47%), 29 (18%), and 54 (35%) patients. There was no significant difference in OS and RFS between single and multiple FLT3 mutations (HR = 0.96, 95% CI: 0.64–1.43, *p* = 0.85; HR = 1.17, 95% CI: 0.75–1.8, *p* = 0.48, for OS and RFS respectively) (Fig. 2a, b). For patients treated with high intensity chemotherapy and TKI, there were no statistical difference in OS and RFS based on the number of *FLT3*-ITD mutations with HR of 0.87 (95% CI: 0.47–1.58, *p* = 0.64), and HR of 0.96 (95% CI: 0.52–1.8, *p* = 0.92) respectively. Similarly for patients treated with low intensity chemotherapy (HR = 1.05 95% CI: 0.61–1.79, *p* = 0.87, HR = 1.47 95% CI: 0.76–2.89, *p* = 0.26 for OS and RFS, respectively).

Moreover, in the single *FLT3* mutant subgroup, the addition of a TKI to high intensity chemotherapy significantly improved OS and RFS compared with patients who did not receive TKI (HR = 0.55, 95% CI: 0.34–0.88, *p* = 0.01, HR = 0.52, 95% CI: 0.31–0.87, *p* = 0.01, for OS and RFS respectively). This beneficial effect of TKI was not statistically different in the multiple *FLT3* mutant subgroup (HR = 0.6, 95% CI: 0.32–1.12, *p* = 0.11, HR = 0.57, 95% CI: 0.3–1.08, *p* = 0.09 for OS and RFS respectively). In patients treated with low intensity chemotherapy, there was no statistical difference in both OS and RFS based on TKI use in both single and multiple mutants.

### Prognostic significance of *FLT3*-ITD length

Among all patients treated with TKI, *FLT3*-ITD length was available on 151 (96%) patients. For patients with multiple *FLT3* mutants, the largest mutant length was accounted for analysis. The median mutant length was 50 bp (range, 7–207 bp). Patients were categorized into long (size ≥50 bp) and short (size <50 bp) *FLT3*-ITD length. There was no significant difference in both OS and RFS according to mutant length (HR = 0.91, 95% CI: 0.61–1.37, *p* = 0.67, and HR = 0.94, 95% CI: 0.6–1.48, *p* = 0.81, for OS and RFS respectively) (Fig. 2c, d). In addition, patients with long *FLT3*-ITD as well short *FLT3*-ITD had similar OS and RFS within their respective group of high or low intensity chemotherapy.

When compared with the no TKI group, patients with long *FLT3*-ITD and treated with high intensity chemotherapy with TKI had a 2-year OS of 58% versus 41% for patients treated without TKI (HR = 0.64, 95% CI: 0.36–1.13, *p* = 0.12). Similarly to the patients with short FLT3-ITD, there was no statistical difference in both OS and RFS (HR = 0.72, 95% CI: 0.41–1.25, *p* = 0.24, HR = 0.65, 95% CI: 0.37–1.15, *p* = 0.14, for OS and RFS respectively).

### Prognostic significance of *FLT3*-ITD allelic ratio

The median *FLT3-*ITD allelic ratio for all patients was 0.51 (range, 0.01–7.7), and those treated with TKI was 0.51 (range, 0.01–2.56). We dichotomized patients into two categories: *FLT3* high (AR ≥ 0.5) and *FLT3* low (AR < 0.5) according to ELN 2017 criteria. Among patients treated with TKI, there was no significant difference in both OS and RFS based on allelic ratio (HR = 0.97, 95% CI: 0.65–1.47, *p* = 0.91, HR = 1.06, 95% CI: 0.68–1.69, *p* = 0.78, respectively) (Fig. 2e, f). Restricting the analysis to patients with intermediate risk cytogenetics who received high intensity chemotherapy with TKI, OS, and RFS between patients with high and low allelic ratio was similar, with HR = 0.9 (95% CI: 0.45–1.8, *p* = 0.78) and HR = 0.94 (95% CI: 0.46–1.89, *p* = 0.86) respectively. Patients who received low intensity chemotherapy with TKI had also similar OS and RFS based on allelic ratio, with HR = 1.15 (95% CI: 0.58–2.30, *p* = 0.67) and HR = 1.26 (95% CI: 0.55–2.8, *p* = 0.58) respectively.

It is worth noting that in patients with intermediate risk cytogenetics treated with high intensity chemotherapy before the TKI era, the median OS for those with high allelic ratio (≥0.5) was 8.5 months compared with 17.6 months for patients with low allelic ratio (<0.5) with HR of 1.66 (95% CI: 1.02–2.69, *p* = 0.04). Similarly, the median OS for patients with high allelic ratio (≥0.5) treated with low intensity chemotherapy without TKI was 8.0 months compared with 12.1 months for patients with low allelic ratio (<0.5) with HR of 1.7 (95% CI: 1.04–2.8, *p* = 0.03).

The addition of TKI was associated with significantly longer RFS and OS in patients with high allelic ratio treated with high intensity chemotherapy, the median OS was 42.4 months in the TKI group versus 12.1 months in the no TKI group (HR = 0.41, 95% CI: 0.25–0.67, *p* < 0.001) (Fig. S4-A). The median RFS was 56.8 months versus 6.2 months for patients treated without TKI (HR = 0.39, 95% CI: 0.23–0.66, *p* < 0.001) (Fig. S4-B). This benefit of adding TKI was not statistically significant in patients with low allelic ratio either in the high intensity or the low intensity chemotherapy group, with HR = 0.71 (95% CI: 0.42–1.22), *p* = 0.18 for OS and HR = 0.70 (95% CI: 0.39–1.23), *p* = 0.19 for RFS in the high intensity chemotherapy group (Fig. S4C-D), and HR = 0.98 (95% CI: 0.59–1.60), *p* = 0.89 for OS in the low intensity chemotherapy group. There was also no benefit from the addition of TKI to low intensity chemotherapy in high allelic ratio group (HR = 0.83, 95% CI: 0.53–1.31, *p* = 0.43 for OS).

### Prognostic significance of *NPM1* co-mutation

**Fig. 3:**
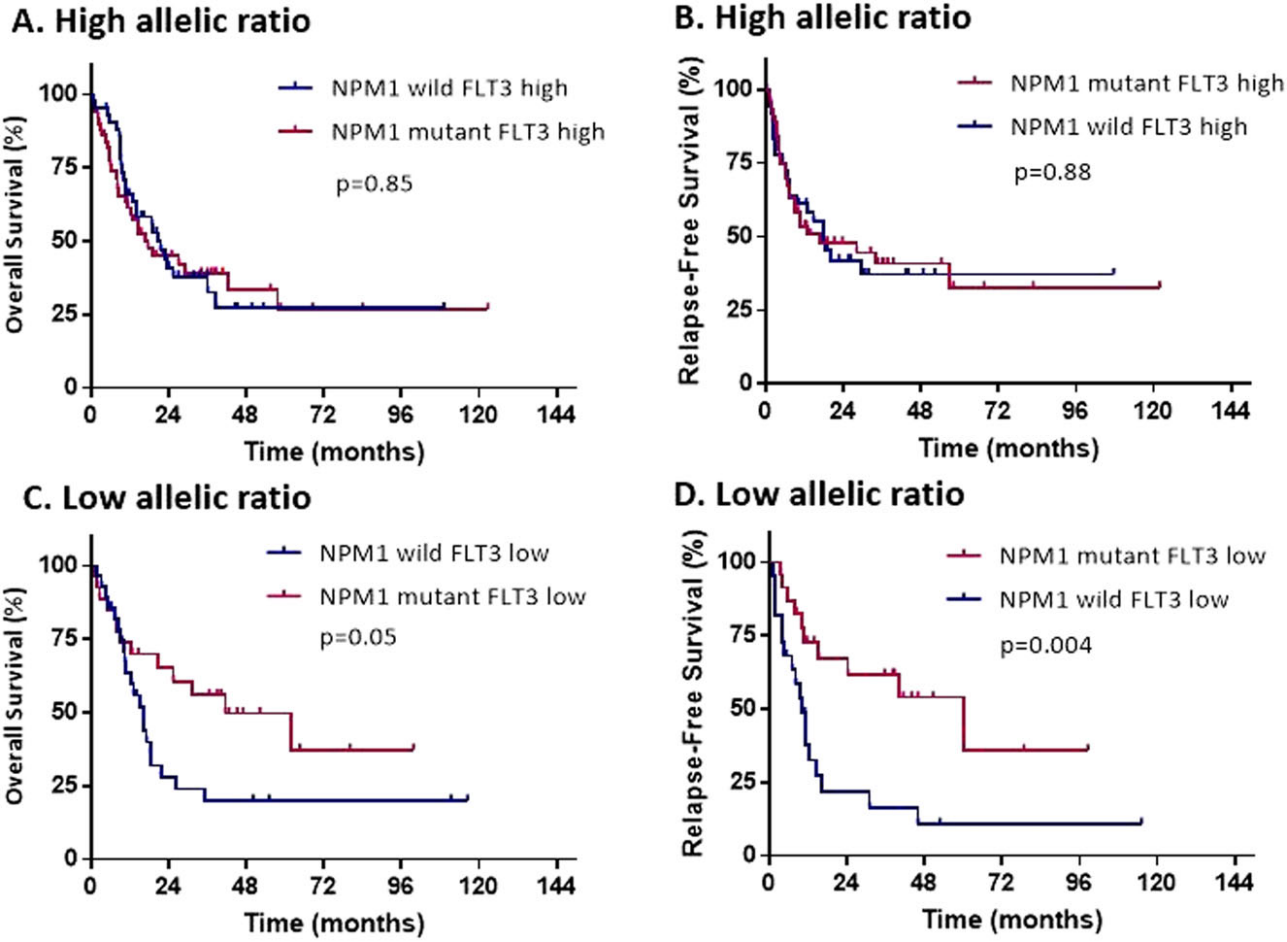
Survival outcomes by NPM1 co-mutation and FLT3 allelic ratio. **a**, **b** Overall survival and relapse free survival in patients treated with TKI regardless of intensity of chemotherapy by NPM1 status within the FLT3 high allelic group. **c**, **d** Overall survival and relapse free survival in patients treated with TKI regardless of intensity of chemotherapy by NPM1 status within the FLT3 low allelic group.

Among all patients treated with a TKI, 147 (94%) patients had an available *NPM1* status. Of them, 77 (52%) patients were *NPM1* mutant. There was no statistical difference in both OS and RFS between *NPM1* wild and *NPM1* mutant groups (HR = 1.24, 95% CI: 0.81–1.88, *p* = 0.31, HR = 1.52, 95% CI: 0.96–2.40, *p* = 0.07, respectively) (Fig. S5A-B). When further stratifying patients according to NPM1 status and FLT3 allelic ratio, those with FLT3 high (≥0.5) had similar OS and RFS whether NPM1 was present or not (HR = 1.05, 95% CI: 0.62–1.79, *p* = 0.85, HR = 0.95, 95% CI: 0.53–1.72, *p* = 0.88, for OS and RFS respectively), whereas patients with FLT3 low (<0.5) had better OS and RFS when NPM1 co-mutation was present (HR = 0.51, 95% CI: 0.26–1.03, *p* = 0.05, HR = 0.32, 95% CI: 0.15–0.73, *p* = 0.004 for OS and RFS respectively) (Fig. 3).

**Fig. 4:**
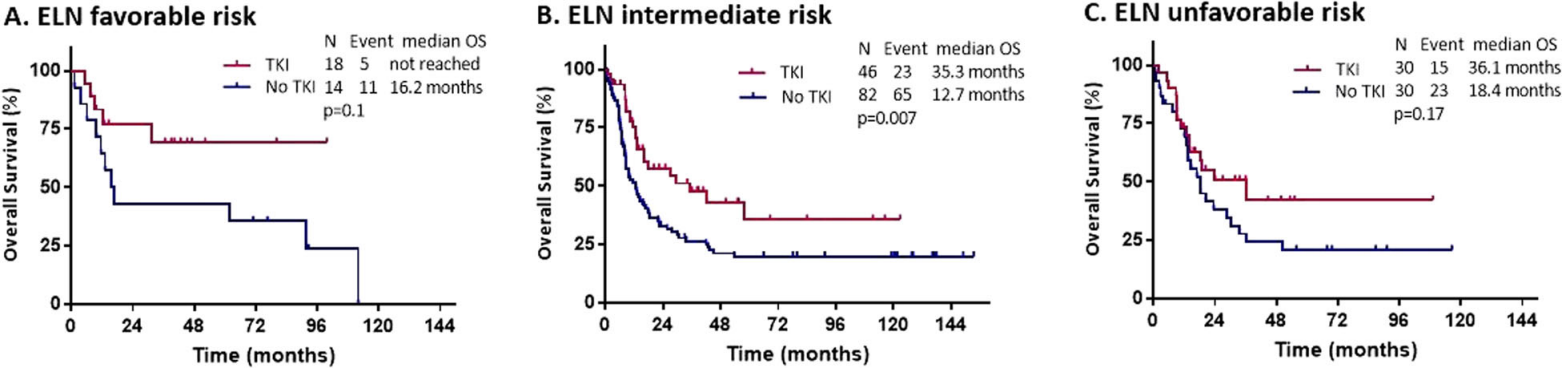
TKI efficacy by ELN criteria. Overall survival in patients treated with higher intensity chemotherapy by TKI use within ELN 2017 risk categories, **(a)** favorable **(b)** intermediate **(c)** unfavorable.

When compared with patients who did not receive TKI, those who were treated with high intensity chemotherapy and TKI had prolonged OS and RFS when NPM1 was present (HR = 0.37, 95% CI: 0.19–0.71, *p* = 0.001, HR = 0.32, 95% CI: 0.17–0.64, *p* < 0.001 for OS and RFS respectively) (Fig. S6). By ELN risk groups, OS and RFS were improved by presence of NPM1 mutation in all subgroups, but it was only statistically different in the intermediate group with HR of 0.53 (95% CI: 0.33–0.85, *p* = 0.007) for OS and HR of 0.62 (95% CI: 0.38–0.99, *p* = 0.05) for RFS (Fig. 4).

### Multivariate analysis

We performed a multivariate analysis including all patients treated with high intensity chemotherapy (*n* = 223). Variables included in the univariate analysis included age, gender, WBC, BM blasts, cytogenetics, *FLT3*-ITD length, *FLT3*-ITD allelic ratio, *FLT3*-ITD numerical variation, co-mutations with *FLT3* TKD point mutations, *NPM1*, *IDH1/2* and *DNMT3A* as well as the use of TKI and alloHSCT as a time-dependent variable. After adjusting for all significant variables, older age ≥65 years, and higher WBC ≥ 20 × 10^9^/L were associated with worse OS (HR = 2, 95% CI: 1.27–3.16, *p* = 0.003, HR = 1.92, 95% CI: 1.37–2.69, *p* < 0.001 respectively), whereas the use of TKI and alloHSCT were associated with significant improvement in OS (HR = 0.66, 95% CI: 0.46–0.97, *p* = 0.03, HR = 0.48, 95% CI: 0.32–0.72, *p* < 0.001, respectively) (Table S2).

Using the multivariate analysis for patients treated with low intensity chemotherapy including the same variables, both multiple co-mutations (≥2) as well as the use of alloHSCT were associated with better OS (HR = 0.57, 95% CI: 0.39–0.85, *p* = 0.01, HR = 0.37, 95% CI: 0.18–0.75, *p* = 0.01) (Table S3).

### Subgroup interaction analysis

In order to identify in which subgroups TKI was largely beneficial, we performed a subgroup interaction analysis and determined the main factors that can contribute to TKI efficacy. The addition of TKI to intensive chemotherapy was associated with significantly better survival mainly in the following subgroups: younger patients <65 years (HR = 0.55, 95% CI: 0.38–0.82, *p* = 0.003), high allelic burden ≥0.5 (HR = 0.41, 95%CI: 0.25–0.66, *p* < 0.001), single ITD mutation (HR = 0.55, 95% CI: 0.34–0.88, *p* = 0.01), diploid cytogenetics (HR = 0.52, 95%CI: 0.35–0.76, *p* = 0.001), NPM1 co-mutation (HR = 0.35, 95% CI: 0.19–0.67, *p* = 0.001) (Tables S4A–S5). In patients treated with low intensity chemotherapy, the addition of TKI did not show any survival benefit including all subgroups (Tables S4B–S6).

## Discussion

In our analysis, the presence of more than one ITD or size of ITD insert did not impact outcome among patients with newly diagnosed FLT3-ITD mutated AML. On the other hand, the addition of TKI including sorafenib or quizartinib to higher intensity chemotherapy was associated with 47% reduction in the risk of death in patients newly diagnosed with *FLT3* mutant AML, taking in consideration that the majority of our patients received triplet combination of induction chemotherapy, unlike ‘doublets’ commonly used in induction regimens. In the RATIFY trial where 717 patients with *FLT3*-ITD and/or TKD mutant AML were randomized to receive midostaurin or placebo in addition to standard intensive chemotherapy, OS was significantly longer with HR of 0.78 and *p* value of 0.009^17^. Based on this trial, the food and drug administration approved midostaurin to be used with intensive chemotherapy for patients with newly diagnosed *FLT3* mutant AML^18^. Midostaurin as well as sorafenib were also effective in older patients (60–70 years) treated with intensive chemotherapy^19,20^. When censored for alloHSCT, we did not find any significant differences in OS and RFS between patients who received TKI and who did not, similarly to what is found in the RATIFY trial^17^.

Lower intensity chemotherapy (HMA or LDAC) has been standard of care in older patients and it was reasonable to add TKI to these agents. Preclinical data also demonstrated synergy between FLT3 inhibition and HMA^21^. In a phase II trial, 27 older patients with untreated *FLT3* mutant AML, median age of 74 years (range, 61–86 years), were treated with 5-azacitidine and sorafenib. Patients experienced favorable overall response rate (ORR) of 78% including a CR rate of 45%, however their median OS was only 8.3 months (range, 1–63 months)^22^. Sorafenib was also added to low dose cytarabine for older patients in a phase I/II trial and resulted in a very low ORR of 10%^23^. In our study, among 62 older patients, median age of 72 years (range, 52–86 years), treated with lower intensity chemotherapy and TKI, 82% of patients received HMAs; 65% received sorafenib; and 21% received quizartinib. For all patients treated with low intensity chemotherapy, the addition of TKI did not improve OS and RFS. This can be explained partly by the inability to administer full doses of sorafenib (most commonly used in our cohort) to the elderly population due to toxicities of myelosuppression, fatigue, etc. However, the combination of HMA and quizartinib, a second generation FLT3 inhibitor, albeit in a small cohort, appears to be promising with a 9 month improvement in median OS, when compared with HMA alone (median OS: 20.4 versus 11.4 months respectively, *p* = 0.034). Initial results of the combination of quizartinib with chemotherapy or HMAs indicates that it is feasible and effective in newly diagnosed older patients with *FLT3* mutant AML^24,25^.

The presence of multiple *FLT3-*ITD mutants did not affect OS and RFS in our total cohort of patients treated with TKI. Several prior reports in the pre-TKI era have found different controversial associations between survival outcomes and the numerical variation of *FLT3* mutations^2,10,26^. Interestingly, we have shown that TKI has a differential effect when added to intensive chemotherapy, in the single mutation rather than multiple mutations. A biological explanation for this difference is unclear. Probably, multiple *FLT3*-ITD mutations are indicative of the presence of multiple AML subclones occurring at different allelic burden and different insertion sites, thus might be differentially sensitive to TKI. Clonal heterogeneity of *FLT3*-ITD manifested by multiple mutations was previously shown to be associated with adverse outcomes^27^.

The impact of *FLT3*-ITD length on the prognosis of patients with *FLT3* mutant AML is still controversial^10–12,28^. Liu et al. reported worse outcomes with longer *FLT3*-ITD with 3-year OS of 27% compared with 61% for short *FLT3*-ITD (*p* = 0.004)^28^. Other reports did not show a significant impact of *FLT3*-ITD insertion length on AML outcomes^10^. Theoretically, longer *FLT3*-ITD has stronger phosphorylation signals and higher constitutive proliferative capacity regardless of the insertion site, reflected by less sensitivity to TKI when AML cell lines were exposed to quizartinib^28^, but this does not seem to be corroborated in the clinical experience, as shown in our analysis.

A high mutant to wild-type allelic ratio of *FLT3*-ITD is known to be associated with poor prognosis due to high relapse risk^3,29^. Samples with a high mutant allelic ratio were more likely to be responsive to FLT3 inhibition compared with samples with low allelic ratio, most likely reflecting an oncogenic addiction to mutant FLT3 for cell survival^30^. Alternatively, AML cells with a lower *FLT3* allelic ratio exhibit a reduced inhibitory effects of FLT3 inhibitors mediated by increased activation of the *FLT3* wild-type^31^. We observed that patients with higher *FLT3* allelic ratio had more pronounced survival benefit when treated with high dose chemotherapy and TKI. The benefit of addition of TKI is less in patients with lower *FLT3* allelic ratio, where *FLT3* mutant clone is not dominant and most likely other mutations are contributing in the evolution of disease. Until now, FLT3 inhibitors are offered to all patients with *FLT3* mutant AML regardless of the allelic ratio. Probably in the near future, incorporation of other targeted agents like BCL-2 inhibitors, MDM2 inhibitors, etc. into the frontline setting will change the current clinical paradigms. Moreover, this differential benefit was more prominent among patients with NPM1 co-mutation. While NPM1 mut/FLT3-ITD low group had the longest survival in the patients treated in RATIFY trial, in a post-hoc analysis of the RATIFY trial, most benefit from addition of midostaurin was shown in the NPM1 wild/FLT3-ITD high group^32^. This discrepancy could be probably explained in part by the differences in induction chemotherapy. In the RATIFY trial, 7 + 3 regimen was used, whereas in our study, 66% of our patients were treated with idarubicin, cytarabine in addition to a nucleoside analog. This triplet combination can produce a higher rate of intracellular cytarabine accumulation, which is preferentially active in *NPM1* mutant AML. However, neither the RATIFY trial, nor our analysis is adequately powered to answer the issue definitively. Moreover, FLT3 inhibition by lestaurtinib appeared to be higher in patients with *NPM1* mutations (81% versus 39% inhibited, *p* = 0.003)^33^. *NPM1* mutations are significantly more frequent in patients with *FLT3*-ITD affecting JMD region and less frequent with insertions located in the beta-1 sheet. There is an increasing evidence that insertions in the beta-1 sheet region are less sensitive to FLT3 inhibitors, explaining our observation of higher beneficial effect of FLT3 inhibitors with *NPM1* co-mutation^34,35^.

In summary, in our cohort of patients who were treated with FLT3 inhibitors, we were unable to find a significant correlation between *FLT3*-ITD numerical variation, mutation length, allelic burden, co-occurring *NPM1* mutation, and clinical outcomes of patients. The addition of FLT3 inhibitors to intensive chemotherapy was associated with better OS and RFS, but not in combination with HMA and low dose cytarabine. The benefit of FLT3 inhibitors was more pronounced in younger patients with high *FLT3* allelic ratio, single mutation, and *NPM1* co-mutation. More research efforts are now underway to incorporate newer generation FLT3 inhibitors to the first line treatment of *FLT3* mutant AML and further improve the prognosis.

## Supplementary information

Supplementary material

## References

[1] Nakao M (1996). Internal tandem duplication of the flt3 gene found in acute myeloid leukemia. Leukemia.

[2] Kottaridis PD (2001). The presence of a FLT3 internal tandem duplication in patients with acute myeloid leukemia (AML) adds important prognostic information to cytogenetic risk group and response to the first cycle of chemotherapy: analysis of 854 patients from the United Kingdom Medical Research Council AML 10 and 12 trials. Blood.

[3] Thiede C (2002). Analysis of FLT3-activating mutations in 979 patients with acute myelogenous leukemia: association with FAB subtypes and identification of subgroups with poor prognosis. Blood.

[4] Schnittger S (2002). Analysis of FLT3 length mutations in 1003 patients with acute myeloid leukemia: correlation to cytogenetics, FAB subtype, and prognosis in the AMLCG study and usefulness as a marker for the detection of minimal residual disease. Blood.

[5] Grafone T, Palmisano M, Nicci C, Storti S (2012). An overview on the role of FLT3-tyrosine kinase receptor in acute myeloid leukemia: biology and treatment. Oncol. Rev..

[6] Griffith J (2004). The structural basis for autoinhibition of FLT3 by the juxtamembrane domain. Mol. Cell.

[7] Frohling S (2002). Prognostic significance of activating FLT3 mutations in younger adults (16 to 60 years) with acute myeloid leukemia and normal cytogenetics: a study of the AML Study Group Ulm. Blood.

[8] Dohner H (2017). Diagnosis and management of AML in adults: 2017 ELN recommendations from an international expert panel. Blood.

[9] O’Donnell MR (2017). Acute Myeloid Leukemia, Version 3.2017, NCCN Clinical Practice Guidelines in Oncology. J. Natl. Compr. Cancer Netw..

[10] Gale RE (2008). The impact of FLT3 internal tandem duplication mutant level, number, size, and interaction with NPM1 mutations in a large cohort of young adult patients with acute myeloid leukemia. Blood.

[11] Stirewalt DL (2006). Size of FLT3 internal tandem duplication has prognostic significance in patients with acute myeloid leukemia. Blood.

[12] Kusec R (2006). More on prognostic significance of FLT3/ITD size in acute myeloid leukemia (AML). Blood.

[13] Kim Y (2015). Quantitative fragment analysis of FLT3-ITD efficiently identifying poor prognostic group with high mutant allele burden or long ITD length. Blood Cancer J..

[14] Schlenk RF (2014). Differential impact of allelic ratio and insertion site in FLT3-ITD-positive AML with respect to allogeneic transplantation. Blood.

[15] Fischer M (2017). Impact of FLT3-ITD diversity on response to induction chemotherapy in patients with acute myeloid leukemia. Haematologica..

[16] Daver N, Schlenk RF, Russell NH, Levis MJ (2019). Targeting FLT3 mutations in AML: review of current knowledge and evidence. Leukemia.

[17] Stone RM (2017). Midostaurin plus chemotherapy for acute myeloid leukemia with a FLT3 Mutation. The New Engl. J. Med..

[18] Levis M (2017). Midostaurin approved for FLT3-mutated AML. Blood.

[19] Schlenk RF (2019). Midostaurin added to chemotherapy and continued single-agent maintenance therapy in acute myeloid leukemia with *FLT3*-ITD. Blood.

[20] Uy GL (2017). A phase 2 study incorporating sorafenib into the chemotherapy for older adults with FLT3-mutated acute myeloid leukemia: CALGB 11001. Blood Adv..

[21] Chang E (2016). The combination of FLT3 and DNA methyltransferase inhibition is synergistically cytotoxic to FLT3/ITD acute myeloid leukemia cells. Leukemia.

[22] Ohanian M (2018). Sorafenib combined with 5-azacytidine in older patients with untreated FLT3-ITD mutated acute myeloid leukemia. Am. J. Hematol..

[23] Macdonald DA (2013). A phase I/II study of sorafenib in combination with low dose cytarabine in elderly patients with acute myeloid leukemia or high-risk myelodysplastic syndrome from the National Cancer Institute of Canada Clinical Trials Group: trial IND.186. Leuk. Lymphoma.

[24] Bowen D (2013). AC220 (Quizartinib) can be safely combined with conventional chemotherapy in older patients with newly diagnosed acute myeloid leukaemia: experience from the AML18 pilot trial. Blood.

[25] Swaminathan M (2017). The combination of quizartinib with azacitidine or low dose cytarabine is highly active in patients (Pts) with FLT3-ITD mutated myeloid leukemias: interim report of a phase I/II trial. Blood.

[26] Borthakur G (2012). Impact of numerical variation in FMS-like tyrosine kinase receptor 3 internal tandem duplications on clinical outcome in normal karyotype acute myelogenous leukemia. Cancer.

[27] Schranz K (2018). Clonal heterogeneity of FLT3-ITD detected by high-throughput amplicon sequencing correlates with adverse prognosis in acute myeloid leukemia. Oncotarget.

[28] Liu SB (2019). Impact of FLT3-ITD length on prognosis of acute myeloid leukemia. Haematologica.

[29] Pratcorona M (2013). Favorable outcome of patients with acute myeloid leukemia harboring a low-allelic burden FLT3-ITD mutation and concomitant NPM1 mutation: relevance to post-remission therapy. Blood.

[30] Pratz KW (2010). FLT3-mutant allelic burden and clinical status are predictive of response to FLT3 inhibitors in AML. Blood.

[31] Chen F, Ishikawa Y, Akashi A, Naoe T, Kiyoi H (2016). Co-expression of wild-type FLT3 attenuates the inhibitory effect of FLT3 inhibitor on FLT3 mutated leukemia cells. Oncotarget.

[32] Döhner K (2017). Prognostic Impact of *NPM1/FLT3-ITD* genotypes from randomized patients with acute myeloid leukemia (AML) treated within the international ratify study. Blood.

[33] Knapper S (2017). A randomized assessment of adding the kinase inhibitor lestaurtinib to first-line chemotherapy for FLT3-mutated AML. Blood.

[34] Rücker FG (2018). Prognostic impact of insertion site in acute myeloid leukemia (AML) with *FLT3* internal tandem duplication: results from the ratify study (Alliance 10603). Blood.

[35] Arreba-Tutusaus P (2016). Impact of FLT3-ITD location on sensitivity to TKI-therapy in vitro and in vivo. Leukemia.

