# Supplementary material for: Impact of numerical variation, allele burden, mutation length and co-occurring mutations on the efficacy of tyrosine kinase inhibitors in newly diagnosed FLT3- mutant acute myeloid leukemia
Source: Blood Cancer J. 2020 May 4;10(5):48. doi: 10.1038/s41408-020-0318-1 (PMC7198530; doi:10.1038/s41408-020-0318-1)
Supplement: Supplementary file 1 — Supplementary material [file 41408_2020_318_MOESM1_ESM.docx]

**Supplementary Material.**

**Impact of numerical variation, allele burden, mutation length and co-occurring mutations on the efficacy of tyrosine kinase inhibitors in newly diagnosed FLT3- mutant acute myeloid leukemia.**

**Tables.**

**Table S1. Frequency of co-occurring mutations.**

| Gene Mutations | Total N= 308 - N (%) |
| --- | --- |
| NPM1 | 142 (46.1) |
| IDH1 / IDH2 | 55 (17.8) |
| DNMT3A | 52 (16.8) |
| RAS | 39 (12.6) |
| CEBPA | 23 (7.5) |
| RUNX1 | 16 (5.2) |
| TET2 | 13 (4.2) |
| PTPN11 | 12 (3.8) |
| TP53 | 6 (1.9) |
| ASXL1 | 5 (1.6) |
| GATA2 | 3 (0.9) |
| EZH2 | 2 (0.6) |
| KIT | 2 (0.6) |

**Table S2. (A) Univariate analysis for OS for patients treated with high dose chemotherapy.**

|  |  | Event/Total | coefficient | HR (95% CI for HR) | P-value |
| --- | --- | --- | --- | --- | --- |
| Age | >=65 years vs. < 65 years | 142/223 | 0.99 | 2.68 (1.72-4.18) | **<.001** |
| Gender | Female vs. Male | 142/223 | 0.17 | 1.19 (0.85-1.66) | 0.31 |
| WBC | >=20 vs. < 20 | 140/221 | 0.72 | 2.06 (1.48-2.88) | **<.001** |
| Diploid cytogenetics | No vs. Yes | 142/220 | -0.17 | 0.85 (0.58-1.23) | 0.38 |
| FLT3-ITD size | Long vs. Short | 102/180 | 0.04 | 1.04 (0.71-1.53) | 0.84 |
| FLT3-ITD ratio | High vs. Low | 140/221 | 0.1 | 1.1 (0.79-1.54) | 0.57 |
| FLT3-ITD No. | multiple vs. single | 140/221 | -0.38 | 0.68 (0.47-0.99) | **0.04** |
| FLT3.D835 | Yes vs. No | 142/223 | 0.29 | 1.33 (0.74-2.4) | 0.34 |
| NPM1 | Yes vs. No | 95/163 | -0.26 | 0.77 (0.52-1.16) | 0.22 |
| IDH 1/2 | Yes vs. No | 59/120 | 0.31 | 1.37 (0.78-2.41) | 0.28 |
| DNMT3A | Yes vs. No | 46/97 | -0.06 | 0.95 (0.49-1.83) | 0.87 |
| No. comutations | >=2 vs. 0 | 125/206 | -0.38 | 0.68 (0.43-1.08) | 0.11 |
|  | 1 vs. 0 |  | -0.27 | 0.77 (0.51-1.15) | 0.20 |
| TKI | Yes vs. No | 142/223 | -0.63 | 0.53 (0.37-0.76) | **<.001** |
| Transplantation | Yes vs. No | 142/223 | -0.97 | 0.38 (0.26-0.56) | **<.001** |

**Table S2. (B) Multivariate** **analysis for OS for patients treated with high dose chemotherapy.**

|  |  | coefficient | HR (95% CI for HR) | P-value |
| --- | --- | --- | --- | --- |
| Age | >=65 years vs. < 65 years | 0.7 | 2 (1.27-3.16) | **0.003** |
| WBC | >=20 vs. < 20 | 0.65 | 1.92 (1.37-2.69) | **<.001** |
| TKI | Yes vs. No | -0.41 | 0.66 (0.46-0.97) | **0.03** |
| Transplantation | Yes vs. No | -0.73 | 0.48 (0.32-0.72) | **<.001** |

**Table S3. (A) Univariate analysis for OS for patients treated with low dose chemotherapy.**

|  |  | Event/Total | coefficient | HR (95% CI for HR) | P-value |
| --- | --- | --- | --- | --- | --- |
| Age | >=65 years vs. < 65 years | 155/172 | 0.01 | 1.01 (0.66-1.55) | 0.96 |
| Gender | Female vs. Male | 155/172 | -0.23 | 0.8 (0.58-1.1) | 0.17 |
| WBC | >=20 vs. < 20 | 155/172 | 0.25 | 1.28 (0.91-1.8) | 0.15 |
| Diploid cytogenetics | No vs. Yes | 143/159 | -0.01 | 0.99 (0.68-1.45) | 0.98 |
| FLT3-ITD size | Long vs. Short | 140/155 | 0.09 | 1.09 (0.78-1.52) | 0.60 |
| FLT3-ITD ratio | High vs. Low | 155/172 | 0.32 | 1.37 (1-1.88) | 0.05 |
| FLT3-ITD No. | multiple vs. single | 155/172 | -0.02 | 0.98 (0.71-1.35) | 0.89 |
| FLT3.D835 | Yes vs. No | 155/172 | 0.03 | 1.03 (0.6-1.76) | 0.91 |
| NPM1 | Yes vs. No | 123/138 | -0.05 | 0.95 (0.66-1.37) | 0.79 |
| IDH 1/2 | Yes vs. No | 101/114 | -0.26 | 0.77 (0.49-1.22) | 0.27 |
| DNMT3A | Yes vs. No | 86/97 | -0.31 | 0.73 (0.44-1.21) | 0.23 |
| No. comutations | >=2 vs. 0 | 147/163 | -0.58 | 0.56 (0.38-0.82) | **0.003** |
|  | 1 vs. 0 |  | -0.18 | 0.83 (0.56-1.25) | 0.38 |
| TKI | Yes vs. No | 155/172 | -0.03 | 0.97 (0.7-1.35) | 0.87 |
| Transplantation | Yes vs. No | 155/172 | -0.88 | 0.41 (0.21-0.8) | **0.01** |

**Table S3. (B) Multivariate analysis for OS for patients treated with low dose chemotherapy.**

|  |  | coefficient | HR (95% CI for HR) | P-value |
| --- | --- | --- | --- | --- |
| No. comutations | >=2 vs. 0 | -0.55 | 0.57 (0.39-0.85) | **0.01** |
|  | 1 vs. 0 | -0.29 | 0.75 (0.5-1.12) | 0.16 |
| Transplantation | Yes vs. No | -1 | 0.37 (0.18-0.75) | **0.01** |

**Table S4. (A) Interaction subgroup analysis for patients treated with high intensity chemotherapy.**

| Covariate |  |  | HR | 2-year OS TKI | 2-year OS No TKI | P value |
| --- | --- | --- | --- | --- | --- | --- |
| Age | < 65 years | 197 (88.34%) | 0.55 (0.38-0.82) | 0.63 | 0.41 | 0.003 |
|  | >=65 years | 26 (11.66%) | 0.67 (0.25-1.8) | - | 0.12 | 0.43 |
| Gender | Female | 127 (56.95%) | 0.5 (0.32-0.79) | 0.6 | 0.32 | 0.003 |
|  | Male | 96 (43.05%) | 0.55 (0.3-0.99) | 0.56 | 0.42 | 0.05 |
| WBC | < 20 | 139 (62.33%) | 0.57 (0.35-0.93) | 0.67 | 0.45 | 0.02 |
|  | >=20 | 82 (36.77%) | 0.52 (0.3-0.92) | 0.39 | 0.26 | 0.03 |
| Diploid cytogenetics | No | 63 (28.25%) | 0.52 (0.25-1.07) | 0.63 | 0.39 | 0.07 |
|  | Yes | 157 (70.4%) | 0.5 (0.33-0.76) | 0.57 | 0.33 | 0.001 |
| FLT3-ITD size | Long | 91 (40.81%) | 0.64 (0.36-1.13) | 0.58 | 0.41 | 0.12 |
|  | Short | 89 (39.91%) | 0.72 (0.41-1.25) | 0.62 | 0.45 | 0.24 |
| FLT3-ITD ratio | High | 114 (51.12%) | 0.41 (0.25-0.66) | 0.63 | 0.36 | <.001 |
|  | Low | 107 (47.98%) | 0.7 (0.41-1.19) | 0.52 | 0.37 | 0.18 |
| FLT3-ITD No. | multiple | 72 (32.29%) | 0.6 (0.32-1.12) | 0.61 | 0.52 | 0.11 |
|  | single | 149 (66.82%) | 0.55 (0.34-0.88) | 0.56 | 0.33 | 0.01 |
| FLT3.D835 | No | 207 (92.83%) | 0.52 (0.36-0.76) | 0.59 | 0.37 | <.001 |
|  | Yes | 16 (7.17%) | 0.66 (0.2-2.19) | 0.5 | 0.3 | 0.50 |
| NPM1 | No | 84 (37.67%) | 0.65 (0.38-1.13) | 0.48 | 0.37 | 0.13 |
|  | Yes | 79 (35.43%) | 0.35 (0.19-0.67) | 0.76 | 0.38 | 0.001 |
| IDH 1/2 | No | 91 (40.81%) | 0.45 (0.24-0.85) | 0.68 | 0.43 | 0.01 |
|  | Yes | 29 (13%) | 0.58 (0.22-1.52) | 0.66 | 0.44 | 0.26 |
| DNMT3A | No | 70 (31.39%) | 0.45 (0.23-0.89) | 0.66 | 0.41 | 0.02 |
|  | Yes | 27 (12.11%) | 0.42 (0.13-1.35) | 0.74 | 0.44 | 0.15 |
| No. comutations | 0 | 88 (39.46%) | 0.84 (0.46-1.52) | 0.37 | 0.38 | 0.56 |
|  | 1 | 62 (27.8%) | 0.42 (0.21-0.82) | 0.69 | 0.36 | 0.01 |
|  | >=2 | 56 (25.11%) | 0.59 (0.27-1.27) | 0.65 | 0.49 | 0.17 |

**Table S4. (B) Interaction subgroup analysis for patients treated with low intensity chemotherapy.**

| Covariate |  |  | HR | 2-year OS TKI | 2-year OS No TKI | P value |
| --- | --- | --- | --- | --- | --- | --- |
| Age | < 65 years | 28 (16.28%) | 0.59 (0.24-1.41) | 0.42 | 0.05 | 0.23 |
|  | >=65 years | 144 (83.72%) | 1.07 (0.75-1.52) | 0.15 | 0.19 | 0.72 |
| Gender | Female | 76 (44.19%) | 1.06 (0.65-1.74) | 0.22 | 0.24 | 0.82 |
|  | Male | 96 (55.81%) | 0.94 (0.6-1.46) | 0.16 | 0.12 | 0.77 |
| WBC | < 20 | 120 (69.77%) | 0.97 (0.65-1.44) | 0.22 | 0.17 | 0.88 |
|  | >=20 | 52 (30.23%) | 0.98 (0.55-1.75) | 0.11 | 0.15 | 0.95 |
| Diploid cytogenetics | No | 42 (24.42%) | 0.95 (0.49-1.85) | 0.14 | 0.15 | 0.88 |
|  | Yes | 117 (68.02%) | 0.81 (0.54-1.21) | 0.23 | 0.13 | 0.30 |
| FLT3-ITD size | Long | 79 (45.93%) | 0.87 (0.55-1.38) | 0.21 | 0.15 | 0.55 |
|  | Short | 76 (44.19%) | 0.98 (0.59-1.63) | 0.17 | 0.19 | 0.94 |
| FLT3-ITD ratio | High | 85 (49.42%) | 0.83 (0.53-1.31) | 0.14 | 0.12 | 0.43 |
|  | Low | 87 (50.58%) | 0.98 (0.59-1.62) | 0.27 | 0.19 | 0.94 |
| FLT3-ITD No. | multiple | 67 (38.95%) | 1.01 (0.61-1.67) | 0.16 | 0.24 | 0.96 |
|  | single | 105 (61.05%) | 0.95 (0.6-1.5) | 0.23 | 0.13 | 0.82 |
| FLT3.D835 | No | 156 (90.7%) | 0.96 (0.68-1.37) | 0.2 | 0.17 | 0.83 |
|  | Yes | 16 (9.3%) | 1.03 (0.37-2.84) | 0.11 | 0.14 | 0.96 |
| NPM1 | No | 75 (43.6%) | 0.87 (0.53-1.42) | 0.19 | 0.14 | 0.57 |
|  | Yes | 63 (36.63%) | 1.28 (0.75-2.18) | 0.21 | 0.29 | 0.37 |
| IDH 1/2 | No | 83 (48.26%) | 0.83 (0.53-1.31) | 0.19 | 0.15 | 0.43 |
|  | Yes | 31 (18.02%) | 1.25 (0.56-2.76) | 0.29 | 0.31 | 0.59 |
| DNMT3A | No | 72 (41.86%) | 0.92 (0.56-1.51) | 0.18 | 0.19 | 0.73 |
|  | Yes | 25 (14.53%) | 2.1 (0.49-9.07) | 0.21 | 0.33 | 0.32 |
| No. comutations | 0 | 64 (37.21%) | 0.57 (0.29-1.13) | 0.18 | 0.04 | 0.11 |
|  | 1 | 42 (24.42%) | 1.22 (0.64-2.32) | 0.13 | 0.24 | 0.54 |
|  | >=2 | 57 (33.14%) | 1.66 (0.91-3.02) | 0.23 | 0.34 | 0.10 |

**Table S5. Subgroup analysis for OS between TKI and no TKI groups (High dose chemotherapy).**


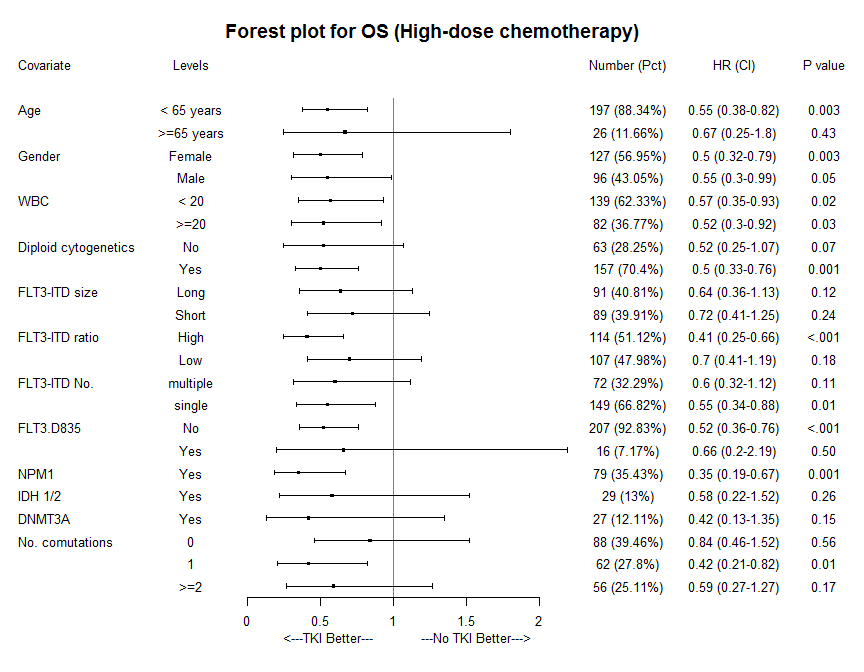


**Table S6. Subgroup analysis for OS between TKI and no TKI groups (low dose chemotherapy).**


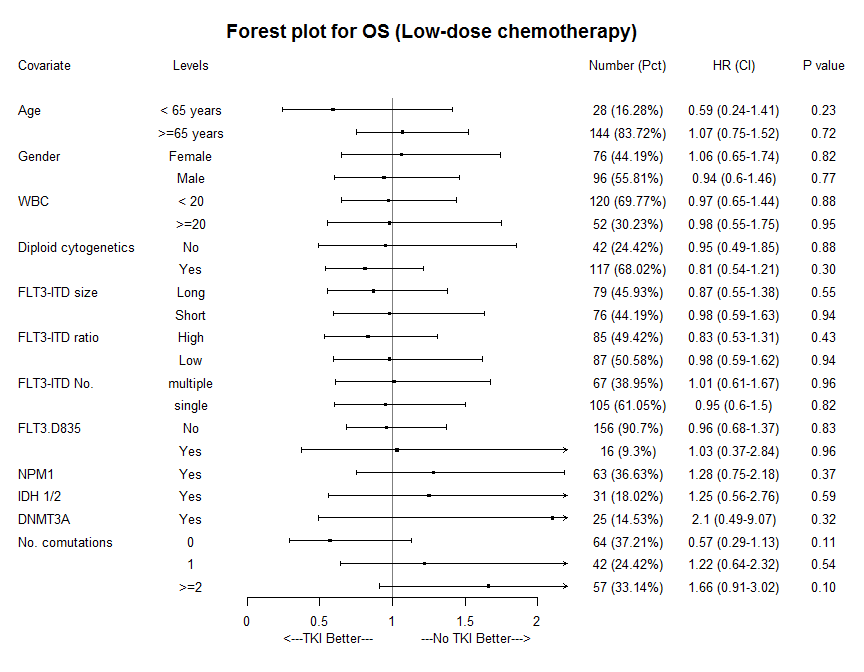


**Figures**.

**Figure S1. Overall survival and relapse free survival in patients treated with high intensity chemotherapy censored for allogeneic hematopoietic stem cell transplantation.**


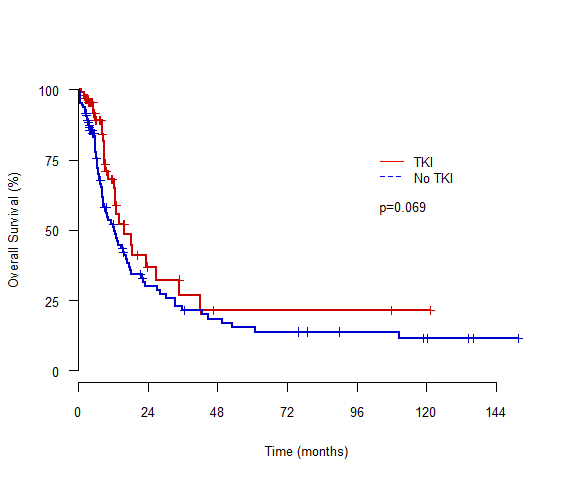

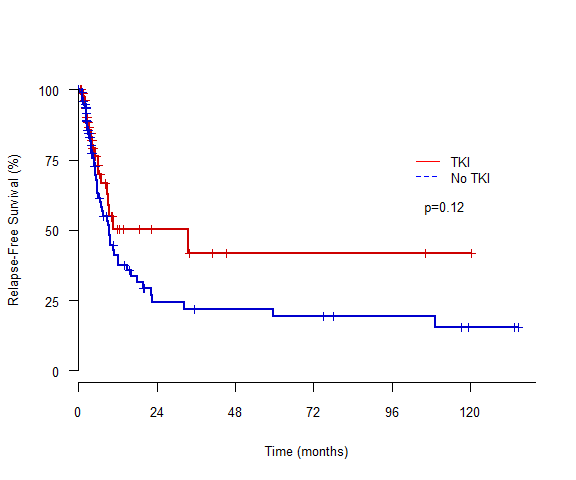


**Figure S2.**

**Figure S3.** Overall survival in patients receiving HMA-based chemotherapy by TKI use (A) quizartinib (B) sorafenib.

**
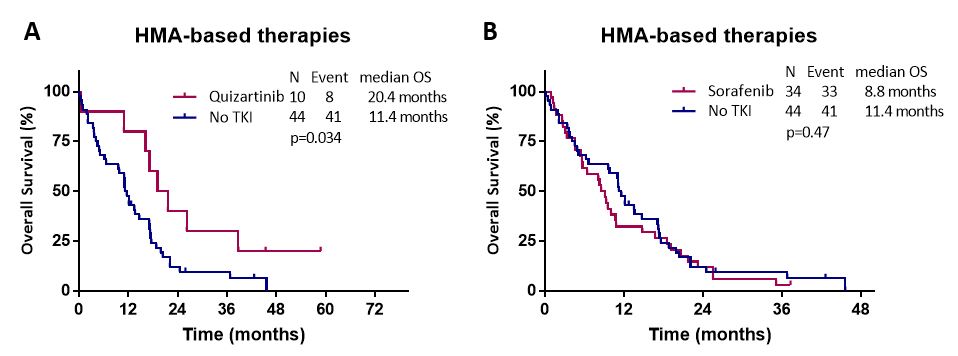
**

**Figure S4. (A-B)** Overall survival and relapse free survival in patients with FLT3 high allelic ratio receiving high dose chemotherapy by TKI use. **(C-D)** Overall survival and relapse free survival in patients with FLT3 low allelic ratio receiving high dose chemotherapy by TKI use.

**
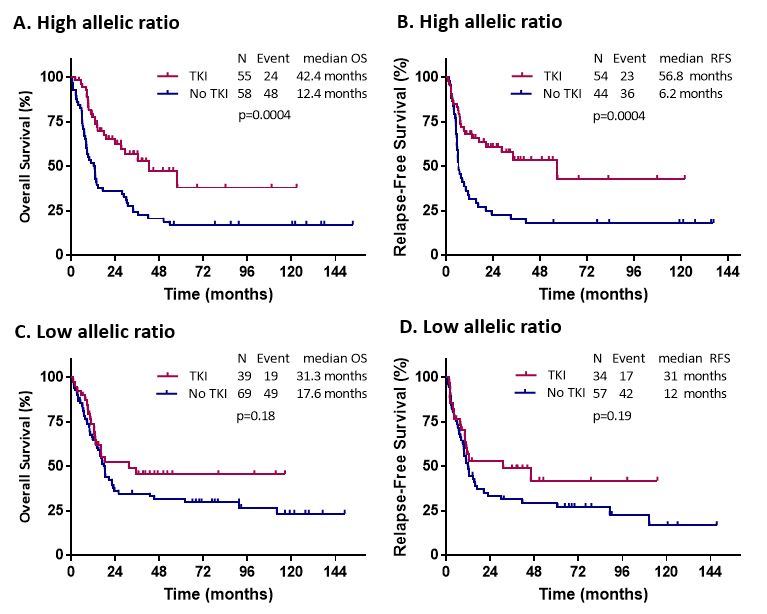
**

**Figure S5. (A-B)** Overall survival and relapse free survival in patients treated with TKI regardless of intensity of chemotherapy by NPM1 status. **(C-D)** Overall survival and relapse free survival in patients treated with TKI regardless of intensity of chemotherapy by ELN 2017 subgroups.

**
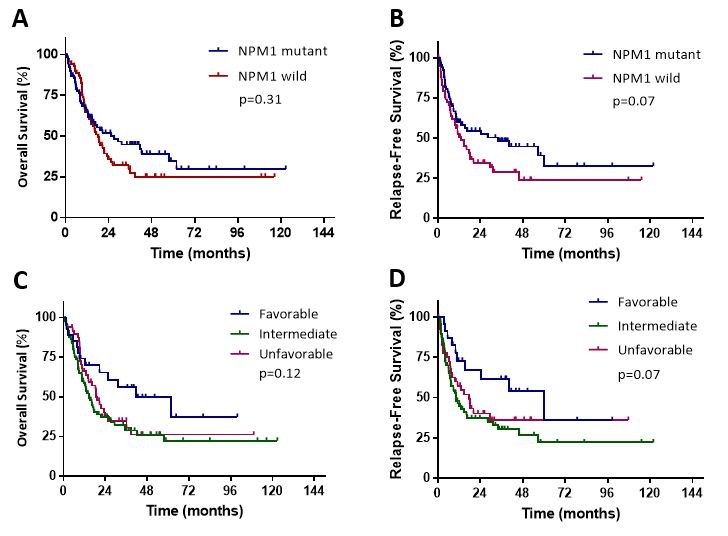
**

**Figure S6.** **(A-B)** Overall survival and relapse free survival in patients treated with high intensity chemotherapy by TKI use within the NPM1 mutant group.

**
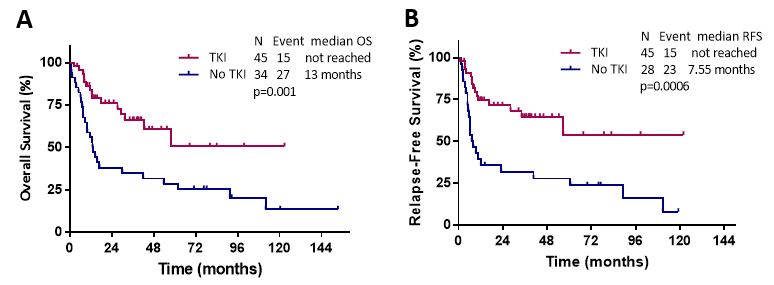
**
